# Mortality in cancer patients with SARS-CoV-2 or seasonal influenza: an observational cohort study from a German-wide hospital network

**DOI:** 10.1007/s15010-022-01852-5

**Published:** 2022-06-03

**Authors:** Cathrin Kodde, Marzia Bonsignore, Daniel Schöndube, Torsten Bauer, Sven Hohenstein, Andreas Bollmann, Andreas Meier-Hellmann, Ralf Kuhlen, Irit Nachtigall

**Affiliations:** 1 Department of Respiratory Diseases “Heckeshorn”, Helios Clinic Emil-Von-Behring, Berlin, Germany; 2 Division of Infectious Diseases and Prevention, Helios Hospitals Duisburg, Duisburg, Germany; 3 Department of Oncology and Hematology, Helios Klinikum Bad Saarow, Bad Saarow, Germany; 4 Heart Center Leipzig at University of Leipzig and Leipzig Heart Institute, Leipzig, Germany; 5 Helios Kliniken, Berlin, Germany; 6 Helios Health, Berlin, Germany; 7 Division of Infectious Diseases and Infection Prevention, Helios Hospital Emil-Von-Behring, Berlin, Germany; 8 Institute of Hygiene and Environmental Medicine, Charité-Universitätsmedizin Berlin, Berlin, Germany

**Keywords:** SARS-CoV-2, COVID-19, Seasonal influenza, Cancer, Mortality, Vaccination

## Abstract

**Purpose:**

At the beginning of the COVID-19 pandemic, SARS-CoV-2 was often compared to seasonal influenza. We aimed to compare the outcome of hospitalized patients with cancer infected by SARS-CoV-2 or seasonal influenza including intensive care unit admission, mechanical ventilation and in-hospital mortality.

**Methods:**

We analyzed claims data of patients with a lab-confirmed SARS-CoV-2 or seasonal influenza infection admitted to one of 85 hospitals of a German-wide hospital network between January 2016 and August 2021.

**Results:**

29,284 patients with COVID-19 and 7442 patients with seasonal influenza were included.

Of these, 360 patients with seasonal influenza and 1625 patients with COVID-19 had any kind of cancer. Cancer patients with COVID-19 were more likely to be admitted to the intensive care unit than cancer patients with seasonal influenza (29.4% vs 24.7%; OR 1.31, 95% CI 1.00–1.73 *p* < .05). No statistical significance was observed in the mechanical ventilation rate for cancer patients with COVID-19 compared to those with seasonal influenza (17.2% vs 13.6% OR 1.34, 95% CI 0.96–1.86 *p* = .09). 34.9% of cancer patients with COVID-19 and 17.9% with seasonal influenza died (OR 2.45, 95% CI 1.81–3.32 *p* < .01). Risk factors among cancer patients with COVID-19 or seasonal influenza for in-hospital mortality included the male gender, age, a higher Elixhauser comorbidity index and metastatic cancer.

**Conclusion:**

Among cancer patients, SARS-CoV-2 was associated with a higher risk for in-hospital mortality than seasonal influenza. These findings underline the need of protective measurements to prevent an infection with either COVID-19 or seasonal influenza, especially in this high-risk population.

## Background

Since the beginning of the COVID-19 pandemic, its clinical course has been compared to the one of seasonal influenza.

With the ongoing pandemic, studies have drawn a clear picture regarding risk factors for a worse outcome of SARS-CoV-2 infection. It is well known that SARS-CoV-2 infection carries an increased risk of adverse outcomes, including admission to the intensive care unit, mechanical ventilation and mortality compared to seasonal influenza in the general population [1–4]. These data also show that COVID-19 patients not only have an increased risk of adverse outcome, it seems to be even more serious for patients with severe underlying diseases like cancer.

Even though seasonal influenza has been known for over a century, studies regarding the clinical outcome for adult patients with cancer and seasonal influenza are scarce. Studies examining immunocompromised patients with seasonal influenza suggest a lower complication rate when compared to that in SARS-CoV-2 cancer patients [5, 6]. Thus, substantial uncertainty regarding the difference between COVID-19 and seasonal influenza remains for high-risk population.

As the COVID-19 pandemic continues and seasonal influenza cases are on the rise during winter season, it is crucial to understand the risk factors and clinical evolution in the most vulnerable population and to prevent infection, especially when vaccinations against both infections are available.

Therefore, this study aims to compare cancer patients with SARS-CoV-2 or seasonal influenza virus infections regarding their clinical course and in-hospital mortality.

## Methods

### Study design

The research was conducted as an observational retrospective cohort study in each of 85 hospitals of the Helios group. Helios is a private company with hospitals located throughout the country and one of the largest providers of inpatient and outpatient care in Germany, including a wide range of hospitals from small community structures to university hospital. The patient mix is representative, because all Helios hospitals are fully covered by all health-care insurance plans.

For the study cohort, we used administrative data that were extracted from QlikView (QlikTech, Radnor, Pennsylvania, USA). We included all patients with a laboratory-confirmed COVID-19 infection (COVID-19 cohort: ICD code U07.1) or an infection due to identified seasonal influenza virus (Influenza cohort: ICD code J10). Included were all patients ≥ 18 years with full inpatient treatment and an admission between January 2016 and August 2021. For the analysis of in-hospital mortality, we excluded cases with discharge due to hospital transfer or unspecified reasons like incomplete data sets (2674/36,726; 7.3% of all patients). The cancer cohort was defined by the presence of any of the three related Elixhauser comorbidities (lymphoma, metastatic cancer, solid tumor). Lab-confirmed Co-infections with COVID-19 infection (ICD codes U07.1, U07.2) and seasonal influenza virus (ICD code J10) were excluded which were 38 patients in the total cohort and one patient in the cancer cohort.

### Statistical analysis

Inferential statistics were based on generalized linear mixed models (GLMM) specifying hospitals as random factor. Effects were estimated with the lme4 package (version 1.1-26) in the R environment for statistical computing (version 4.0.2, 64-bit build). In all models, we specified varying intercepts for the random factor hospitals. For all tests, we apply a two-tailed 5% error criterion for significance (*p *value). For the description of the cohorts, we used *χ*^2^ tests for categorical and analysis of variance for continuous variables. The univariate analysis of hospital treatment and in-hospital mortality is based on logistic GLMMs with logit link function. We report proportions, counts, odds ratios, confidence intervals (CI), and *p* values for these models. For in-hospital mortality, we computed multivariable logistic GLMMs with sex, age, and Elixhauser comorbidity index (ECI) as covariates. Dichotomous variables were coded as 0.5 vs  0.5, while age was scaled to zero mean and unit variance.

## Results

A total number of 36726 patients with either seasonal influenza or COVID-19 were admitted to one of 85 hospitals of the Helios-Network in Germany between January 2016 and August 2021. 29284 of these were tested positive for SARS-CoV-2 and 7,442 patients for seasonal influenza. Among the 1,985 cancer patients included, 1625 were tested positive for SARS-CoV-2 and 360 for seasonal influenza.

The gender distribution was not significant different in both infections with a male predominance (COVID-19: 58.2% vs seasonal influenza: 56.1%, *p* = 0.50). However, a lower male predominance was seen in the total cohort (COVID-19: 52.1% males vs. 50.1% males, *p* < 0.01). The baseline characteristics of the cohorts are shown in Table 1.

**Table 1:** Baseline characteristics of the cancer and total cohort

Cancer cohort Proportion (*n*)				Total cohort Proportion (*n*)		
Group	Influenza	COVID-19	*P* value	Influenza	COVID-19	*P* value
Age						
Mean (SD)	70.8 ± 11.8	72.2 ± 11.8	0.04	67.0 ± 18.2	68.0 ± 17.6	< 0.01
18 – 59 years	17.8% (64)	14.3% (233)	0.12	29.4% (2,186)	29.2% (8,544)	0.75
60 – 69 years	24.7% (89)	24.2% (393)	0.88	16.5% (1,231)	16.7% (4,885)	0.79
70 –79 years	30.8% (111)	30.0% (488)	0.81	24.7% (1,837)	21.5% (6,288)	< 0.01
≥ 80 years	26.7% (96)	31.4% (511)	0.09	29.4% (2,188)	32.7% (9,567)	< 0.01
Sex						
Male	56.1% (202)	58.2% (946)		50.1% (3,728)	52.1% (15,269)	
Female	43.9% (158)	41.8% (679)	0.50	49.9% (3,714)	47.9% (14,015)	< 0.01
Elixhauser comorbidity index						
Mean (SD)	23.3 ± 12.6	25.5 ± 13.3	< 0.01	10.4 ± 10.7	11.0 ± 11.5	< 0.01
< 0	0.6% (2)	0.3% (5)	0.82	12.4% (920)	13.6% (3,968)	< 0.01
0	0.0% (0)	0.1% (2)	1.00	14.6% (1,090)	15.6% (4,555)	0.05
01. Apr	1.1% (4)	2.6% (42)	0.14	7.2% (539)	5.4% (1,568)	< 0.01
≥ 5	98.3% (354)	97.0% (1,576)	0.22	65.7% (4,893)	65.5% (19,193)	0.75
Elixhauser comorbidity index (without cancer)						
Mean (SD)	12.5 ± 10.3	13.3 ± 11.4	0.23	9.9 ± 10.2	10.3 ± 10.8	< 0.01
< 0	7.8% (28)	10.3% (168)	0.17	12.7% (946)	14.1% (4,131)	< 0.01
0	10.0% (36)	8.2% (134)	0.33	15.1% (1,126)	16.0% (4,687)	0.07
01. Apr	6.9% (25)	6.2% (101)	0.69	7.5% (560)	5.6% (1,627)	< 0.01
≥ 5	75.3% (271)	75.2% (1,222)	1.00	64.6% (4,810)	64.3% (18,839)	0.64
Congestive heart failure						
Yes	22.2% (80)	24.7% (402)	0.35	22.6% (1,684)	23.8% (6,973)	0.03
Cardiac arrhythmias						
Yes	25.8% (93)	27.4% (445)	0.59	25.8% (1,922)	25.8% (7,557)	0.98
Valvular diseases						
Yes	8.9% (32)	9.0% (147)	1.00	8.0% (595)	7.6% (2,240)	0.33
Pulmonary circulation disorders						
Yes	5.0% (18)	6.5% (105)	0.36	4.0% (300)	5.3% (1,556)	< 0.01
Peripheral vascular disorders						
Yes	13.9% (50)	15.9% (259)	0.37	6.8% (506)	7.6% (2,211)	0.03
Hypertension, uncomplicated						
Yes	37.5% (135)	45.1% (733)	0.01	38.9% (2,893)	44.5% (13,030)	< 0.01
Hypertension, complicated						
Yes	11.9% (43)	13.5% (219)	0.49	13.9% (1,033)	12.5% (3,671)	< 0.01
Paralysis						
Yes	3.6% (13)	5.4% (88)	0.20	4.0% (295)	4.7% (1,389)	< 0.01
Other neurological disorders						
Yes	7.2% (26)	7.9% (128)	0.76	8.2% (613)	8.3% (2,435)	0.85
Chronic pulmonary disease						
Yes	22.8% (82)	14.8% (241)	< 0.01	20.1% (1,496)	12.0% (3,515)	< 0.01
Diabetes, uncomplicated						
Yes	14.2% (51)	18.5% (301)	0.06	14.1% (1,053)	17.6% (5,162)	< 0.01
Diabetes, complicated						
Yes	8.9% (32)	11.4% (186)	0.19	10.1% (748)	11.6% (3,404)	< 0.01
Hypothyroidism						
Yes	12.2% (44)	13.7% (223)	0.50	11.2% (832)	13.0% (3,801)	< 0.01
Renal failure						
Yes	34.4% (124)	34.4% (559)	1.00	28.7% (2,135)	30.1% (8,811)	0.02
Liver disease						
Yes	5.6% (20)	7.1% (116)	0.34	4.1% (305)	4.3% (1,252)	0.52
Peptic ulcer disease excluding bleeding						
Yes	0.0% (0)	0.3% (5)	0.64	0.1% (5)	0.1% (23)	0.93
AIDS/HIV						
Yes	0.0% (0)	0.0% (0)		0.1% (4)	0.0% (9)	0.55
Lymphoma						
Yes	25.6% (92)	12.1% (196)	< 0.01	1.2% (92)	0.7% (196)	< 0.01
Metastatic cancer						
Yes	29.2% (105)	38.7% (629)	< 0.01	1.4% (105)	2.1% (629)	< 0.01
Solid tumor without metastasis						
Yes	74.2% (267)	85.6% (1,391)	< 0.01	3.6% (267)	4.8% (1,391)	< 0.01
Rheumatoid arthritis/collaged vascular disease						
Yes	2.8% (10)	1.3% (21)	0.07	2.2% (164)	1.8% (537)	0.04
Coagulopathy						
Yes	11.1% (40)	10.5% (171)	0.82	4.4% (324)	5.6% (1,644)	< 0.01
Obesity						
Yes	9.4% (34)	11.0% (179)	0.44	11.8% (878)	13.8% (4,050)	< 0.01
Weight loss						
Yes	23.1% (83)	25.4% (413)	0.39	8.8% (658)	11.5% (3,367)	< 0.01
Fluid and electrolyte disorders						
Yes	41.7% (150)	48.6% (789)	0.02	45.0% (3,349)	46.0% (13,466)	0.13
Blood loss anemia						
Yes	1.1% (4)	1.2% (19)	1.00	0.7% (51)	0.5% (149)	0.08
Deficiency anemia						
Yes	2.8% (10)	3.3% (54)	0.72	2.9% (215)	3.4% (1,005)	0.02
Alcohol abuse						
Yes	2.2% (8)	2.3% (38)	1.00	2.7% (200)	1.8% (541)	< 0.01
Drug abuse						
Yes	0.6% (2)	0.2% (4)	0.66	0.6% (46)	0.4% (103)	< 0.01
Psychoses						
Yes	0.6% (2)	0.4% (7)	1.00	1.2% (86)	1.1% (323)	0.75
Depression						
Yes	3.3% (12)	7.5% (122)	< 0.01	5.6% (419)	6.2% (1,830)	0.05
Cancer						
Yes	100.0% (360)	100.0% (1,625)		4.8% (360)	5.5% (1,625)	0.02

COVID-19 cancer patients had significantly more comorbidities seen in a higher Elixhauser index (ECI mean ± SD = 25.5 ± 13.3 vs 23.3 ± 12,6 *p* < 0.01) than the total cohort. Cancer patients with COVID-19 had more often uncomplicated hypertension, renal failure, coagulopathy, diabetes and obesity. Seasonal influenza patients with cancer were statistically more likely to have chronic pulmonary disease (22.8% vs. 14.8%, *p* < 0.01) than COVID-19 patients with cancer. The same difference was also seen in the total cohort.

The clinical outcome after admission differed significantly between cancer patients with COVID-19 or seasonal influenza. SARS-CoV-2-positive patients were more likely to be transferred to ICU (OR 1.31 95% CI 1.00–1.73 *p* = 0.05) and tended to be treated on average 2 days longer in the ICU than patients with seasonal influenza (mean length of 9.3 days vs 7.2 days, 95% CI 0.21; −0.05 to 0.47 *p* = 0.12). COVID-19 cancer patients had a significant higher risk of mortality compared to seasonal influenza patients.

In the cancer cohort, 34.9% of all COVID-19 cancer patients died compared to 17.9% of seasonal influenza patients. This resulted in 2.45 higher odds of in-hospital mortality for COVID-19 patients (OR 2.45 95% CI 1.81–3.32 *p* < 0.01). In the general cohort, the in-hospital mortality was higher in patients with COVID-19 (20.3%) with relative odds of 3.19 (95% CI 2.89–3.51 *p* < 0.01) compared to seasonal influenza patients (7.2%).

Cancer did not increase the risk of each reported adverse outcomes: in the total cohort, 18.8% of SARS-CoV-2-positive patients (5515/29,284) were mechanically ventilated, compared to 11% (816/7442) of patients with seasonal influenza (OR 1.86, 95% CI 1.71–2.02 *p* < 0.01) (Table 2).

**Table 2:** Univariate analysis of outcomes and treatments in the total cohort

Cohort	Proportion (*n*)	Odds ratio (95% CI)	*P* value
Intensive care			
Influenza	21.1% (1,570)		
COVID-19	27.6% (8076)	1.45 (1.36–1.55)	< 0.01
Mechanical ventilation			
Influenza	11.0% (816)		
COVID-19	18.8% (5515)	1.86 (1.71–2.02)	< 0.01
In-hospital mortality*			
Influenza	7.2% (509)		
COVID-19	20.3% (5,484)	3.19 (2.89–3.51)	< 0.01

*Based on 34,052 cases (92.7%). We excluded cases with discharge due to hospital transfer or unspecified reason

In the cancer cohort, 17.2% of COVID-19 patients and 13.6% of seasonal influenza patients were mechanically ventilated. Being tested positive for COVID-19 resulted in a trend for a higher risk of mechanical ventilation (OR 1.34, 95% CI 0.96–1.86 *p* = 0.09). No difference in the duration of mechanical ventilation was observed comparing COVID-19 and seasonal influenza (Table 3).

**Table 3:** Univariate analysis of outcomes and treatments in the cancer cohort

Cohort	Proportion (*n*)	Odds ratio (95% CI)	*P* value
Intensive care			
Influenza	24.7% (89)		
COVID-19	29.4% (477)	1.31 (1.00–1.73)	0.05
Mechanical ventilation			
Influenza	13.6% (49)		
COVID-19	17.2% (280)	1.34 (0.96–1.86)	0.09
In-hospital mortality*			
Influenza	17.9% (61)		
COVID-19	34.9% (530)	2.45 (1.81–3.32)	< 0.01

*Based on 1,858 cases (93.6%). We excluded cases with discharge due to hospital transfer or unspecified reason

**Fig. 1:**
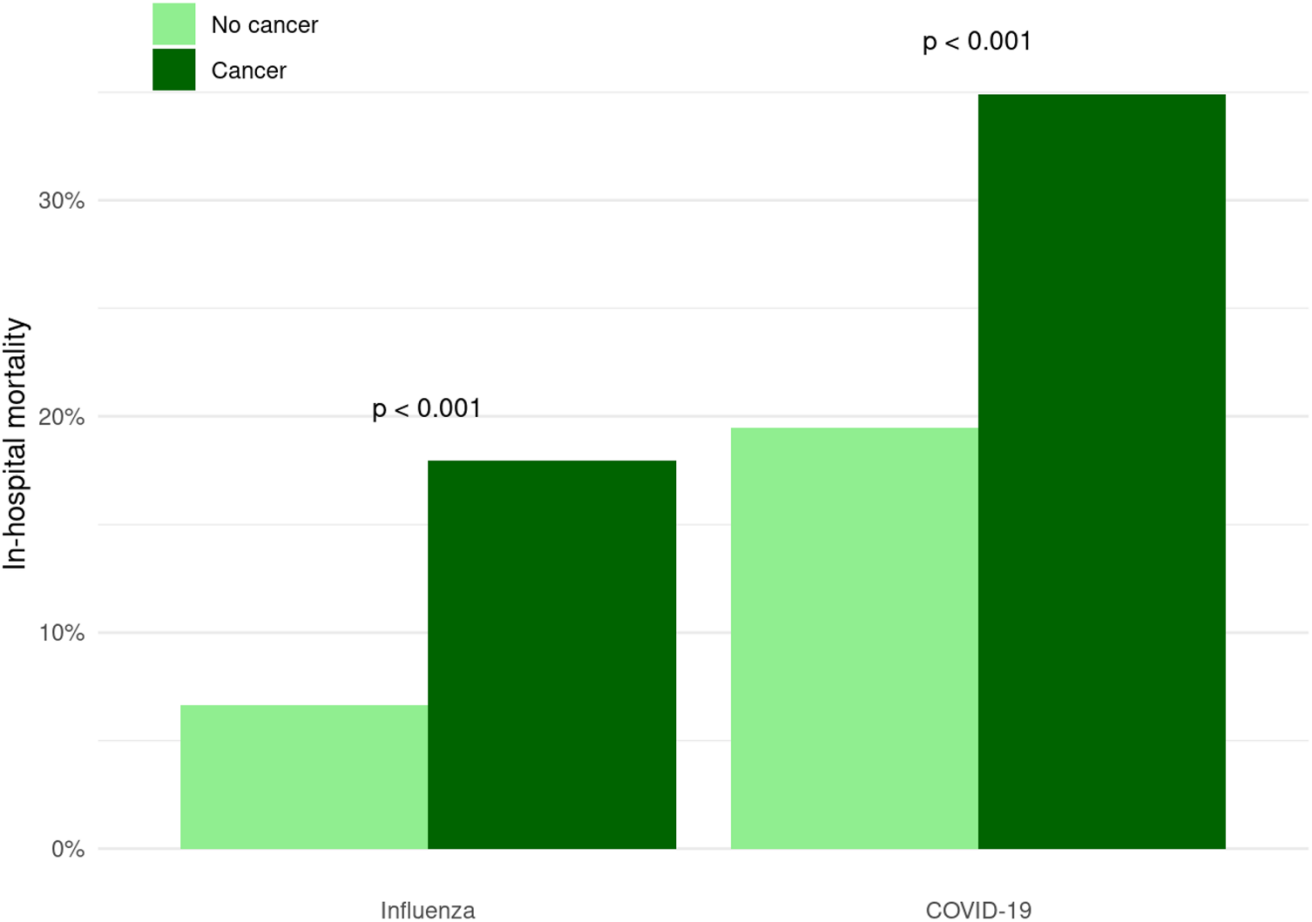
In-hospital mortality as a function of cohort and presence of cancer

An analysis restricted to in-hospital care for those in the COVID-19 group comparing cancer to non-cancer patients showed that cancer patients were less likely to be mechanically ventilated (OR 0.82 95% CI  0.72–0.94 *p* < 0.01). However, they have 2.10 higher odds of dying because of COVID-19.

(OR 2.10, 95% CI 1.88–2.35 *p* < 0.01) and even 3.01 higher odds of mortality because of seasonal influenza (OR 3.01, 95% CI 2.24–4.05 *p* < 0.01). The relative increase of in-hospital mortality for SARS-CoV-2-positive cancer patients is the same as that for patients with seasonal influenza (Fig. 1). Multivariable analysis of in-hospital mortality among the cancer cohort identified different risk factors. Male gender, advanced age, metastatic cancer, COVID-19 in comparison to seasonal influenza and a high ECI were independently associated with statistical significant risk of in-hospital mortality. Interactions between different malignancies (lymphoma, metastatic cancer and solid tumor) and the difference between COVID-19 and influenza were not significant, indicating comparable impacts of different cancer types for COVID-19 and seasonal influenza patients with cancer (Table 4). A multivariable analysis of the total cohort showed that cancer and COVID-19 are an independent risk factor of in-hospital mortality (Table 5).

**Table 4:** Results of multivariable analysis of in-hospital mortality for cancer cohort

Variable	OR (95% CI)	*P* value
Male sex	1.32 (1.06–1.64)	0.01
Age	1.03 (1.01–1.04)	< 0.01
Elixhauser comorbidity index (without cancer)	1.05 (1.04–1.06)	< 0.01
Lymphoma (C81–C85, C88, C96, C90.0, C90.2)	1.79 (0.77–4.15)	0.17
Metastatic cancer (C77–C80)	2.26 (1.59–3.20)	< 0.01
Solid tumor without metastasis (C00–C26, C30–C34, C37–C41, C43, C45–C58, C60–C76, C97)	1.02 (0.45–2.28)	0.97
COVID-19 vs. influenza	2.67 (1.77–4.02)	< 0.01
Interaction COVID-19 × lymphoma	0.73 (0.14–3.90)	0.71
Interaction COVID-19 × metastatic cancer	0.69 (0.34–1.37)	0.29
Interaction COVID-19 × solid tumor	0.45 (0.09–2.25)	0.33

*Based on 1,858 cases (93.6%). We excluded cases with discharge due to hospital transfer or unspecified reason. This cohort is defined by the presence of any of the three related Elixhauser comorbidities (lymphoma, metastatic cancer, solid tumor)

**Table 5:** Results of multivariable analysis of in-hospital mortality for total cohort

Variable	OR (95% CI)	*P* value
Male sex	1.63 (1.53–1.74)	< 0.01
Age	1.06 (1.06–1.06)	< 0.01
Elixhauser comorbidity score (without cancer)	1.06 (1.05–1.06)	< 0.01
COVID-19 vs. influenza	2.98 (2.52–3.54)	< 0.01
Cancer	2.24 (1.90–2.65)	< 0.01
Interaction COVID-19 × cancer	0.68 (0.48–0.95)	0.02

*Based on 34,052 cases (92.7%). We excluded cases with discharge due to hospital transfer or unspecified reason

In-hospital mortality as a function of cohort and presence of cancer

## Discussion

The major finding of this study was that the in-hospital mortality of SARS-CoV-2 and seasonal influenza was markedly increased when patients had cancer. In addition, the in-hospital mortality of SARS-CoV-2 patients was higher with and without cancer in comparison with the corresponding patient with seasonal influenza.

In total, we identified 7442 seasonal influenza and 29,284 COVID-19 cases. Although the observation time for COVID-19 lasted only one-and-a-half years, the number of cases was almost four times higher compared to seasonal influenza over a 5-year period. These findings are consistent with prior studies. It is assumed that the previous seasonal influenza infections lead to partial immunity and that the vaccination against influenza has an additional protective effect, although only 38% of all > 60 years old are vaccinated against influenza in Germany [1, 7, 8]. SARS-CoV-2 was able to hit an immunological naïve population worldwide, thus explaining the severity.

Recent studies showed that the SARS-CoV-2 vaccination that started in December 2020 had a positive impact on the hospital admission rate. This is especially seen when comparing the outcome of the first wave to the others [9, 10].

In comparing studies, hospitalized patients with seasonal influenza have been shown to have more comorbidities and be in tendency older, whereas COVID-19 patients were mainly men, younger with less comorbidities [1, 3, 8, 11, 12]. In our study, COVID-19 patients of the total cohort had a significant higher ECI than seasonal influenza patients, indicating a higher prevalence of comorbidities. This shift may be explained by the fact that previous studies examined mainly the first wave of COVID-19 (December 2019–May 2020). At the beginning of the pandemic, especially in Europe, people of younger/middle age were more affected due to travel and social gatherings, which was an initial accelerator for the pandemic [13]. This changed toward the second surge, where outbreaks in nursing homes and long-term care facilities occurred regularly, leading to a higher median age of patients with more comorbidities. Among other reasons, this initial high infection rate is due to the susceptibility of an immunologically naïve population. Seasonal influenza vaccination, which is recommended for all person ages 60 years and older or with pre-existing conditions in Germany, may have had a protective effect in the elder age group leading to the lower median age in patients with seasonal influenza.

Cancer patients had an overall higher ECI in both COVID-19 and seasonal influenza cases compared to patients with these infections and no cancer. Recent studies addressing both infections showed that cancer patient had in general higher amount of comorbidities because of the cancer itself, its late onset and cancer-related morbidity [4, 14, 15]. In recent studies, diabetes, hypertension and obesity were among the most described comorbidities in both the general and cancer population, which is also seen in our study. Obesity and diabetes are generally known to cause a subtle chronic inflammation and an altered immune system, thus making these patients prone to a severe clinical course of infections [16, 17]. COVID-19 patients were more obese with diabetes presenting COVID-19-associated comorbidities, such as coagulopathy and pulmonary circulation disorders, compared to patients with seasonal influenza. Interestingly, we found in our study that seasonal influenza patients were affected significantly more often from chronic pulmonary disease than COVID-19 patients.

This is in line with previous reports where chronic pulmonary disease was more often found in patients tested positive for seasonal influenza. The fact that chronic respiratory disease is frequently observed in seasonal influenza patients, which have a less severe clinical outcome, supports the assumption of an intrinsic inflammation caused by SARS-CoV-2 leading to its more severe clinical evolution [1, 16–18].

Cancer patients with either one of the infections were older compared to the full cohort. These findings are consistent with studies that described an advanced median age for COVID-19 and seasonal influenza cases compared to the total cohort due to usual late onset of cancer [14, 15, 19, 20]. In the cancer cohort, we saw a male predominance regarding COVID-19 (58.2%) and seasonal influenza (56.1%). In the total cohort, the difference was not so distinct (52.1% vs 50.1%) which is in line with previously published studies [4].

Studies have already shown that COVID-19 in general has a severe outcome with high mortality of mechanically ventilated patients. Even though intensive care treatment and full life-support are widely available in Germany. Many patients, especially those with underlying conditions, died. Malignancies and its therapy can alter the immune system, making patients with cancer prone to a severe course of infections, especially COVID-19.

In our study, COVID-19 cancer patients were more likely to be admitted to the ICU than seasonal influenza patients. The available data for cancer patients with seasonal influenza regarding ICU admission are scarce. The ICU admission rate is described to be 17.6–22.8% in immunocompromised (including cancer) patients [5, 21]. This described COVID-19 ICU rate is consistent with a German-wide study from Rühthrich et al., but it varies widely between countries ranging from 7 to 19% [2, 14, 20, 22, 23]. Studies from Germany generally showed a higher ICU admission rate in cancer patients for COVID-19 compared with other countries, which is probably mainly due to higher ICU bed-capacity in Germany and thus less admission restriction [4, 24, 25]. This finding correlates with the higher mechanical ventilation rate observed in our COVID-19 cancer cohort. We have seen a lower mechanical ventilation rate (13.6%) in cancer patients affected with seasonal influenza than COVID-19 patients (17.2%) without statistical significance. Previous studies reported ventilation rates of COVID-19 patients ranging between 8 and 12% [2, 14, 22, 26, 27], which is lower than the rate that we detected in patients with COVD-19 and cancer. Throughout it is described that the COVID-19 ventilation rate is high with a range from 8 to 12%, but it remains lower than in our study.

In our study, SARS-CoV-2-positive patients had a remarkably higher in-hospital mortality than patients with seasonal influenza. The observed mortality of COVID-19 cancer patients was 34.9%, which is approximately two times higher compared to 17.9% for seasonal influenza, approximately three times higher than in non-cancer patients. This higher mortality in cancer COVID-19 patients is in accordance with other studies reporting a death rate of 21–33% [2, 4, 20, 28]. Especially patients with the diagnosis of lymphoma, including blood malignancies were at special risk of in-hospital mortality. This finding is in accordance to recent studies showing an increased risk due to alternated immune system making them more susceptible for severe course of infections [29].

Our mortality rate is found at the upper end of the published mortality range. A possible explanation could be the long observation time until August 2021. Early studies of the pandemic with a limited number of cases showed a general lower mortality with a study time of only a few months including only partially the second wave [8, 12, 25, 30]. Patients were younger in the early beginning of the pandemic with less comorbidities. In Germany, the mortality rate reached its peak in December 2020 [31], when elderly people living in long-term care facilities were affected. A declining mortality rate has been observed since then. The most recent studies examining the second and third wave of COVID-19 pandemic (when vaccination were not widely available) showed that the mortality and mechanical ventilation rate have declined and tend to be even lower than in seasonal influenza [32, 33].

Even though detailed studies are scarce, an explanation for this development probably is the vaccination programs, which prioritized the most vulnerable —elder— population first. Thus, the intermittent high mortality declined, thanks to public health programs and a better understanding of the disease and possible treatments. However, future studies will show if the mortality rate may be equal or even lower compared to seasonal influenza.

In summary, the increased mortality in the cancer cohort for both COVID-19 and seasonal influenza makes them a high-risk population. Public Health measurements need to address directly this patient group and take action to prevent an infection with either of these deadly diseases.

By promoting vaccination, especially with regard to the higher incidence of viral infection during the colder season, mortality might be reduced.

## Strengths and limitations

Helios is a privately owned hospital network with hospitals spread throughout Germany, including small community hospitals, tertiary care and university hospitals. Because of this wide range and a high number of included patients, a heterogeneity of cases is obtained. Still there remain limitations; tertiary care and university hospitals may be underrepresented resulting in a bias toward less severe cases, and generalizability is limited because of the focus on German patients who were older than 18 years [4].

Especially in patients with advanced cancer, a living will is frequently present. This often includes directives such as a “do not intubate” order. In our study, this might have influenced the rate of invasive ventilation, ICU and mortality.

Another limitation is the fact that we do not know if the patients were diagnosed and hospitalized for COVID-19 or seasonal influenza or if they were just tested positive. Additionally, during the SARS-CoV-2 pandemic, hospitals established testing rules, and almost every patient was tested for SARS-CoV-2 upon admission. This bias may result in an underrepresentation of seasonal influenza cases as not every patient was tested for seasonal influenza.

A limitation is also the direct comparison of seasonal influenza and SARS-CoV-2. Even though they are both viral infections, the transmissibility and pathogenesis differ. Additionally, the population tends to have a partial immunity against seasonal influenza either through a vaccination or through previous infections. SARS-CoV-2 was able to infect a naïve population and thus leading to more severe outcomes [8]. A substantial proportion (7.3%) of patients was excluded from analysis due to transfer to another facility or unspecified reasons like incomplete data. This may have caused a bias.

To our knowledge, this is the first large study to compare clinical characteristics and outcome of SARS-CoV-2 and seasonal influenza infection in patients with cancer. This observational multi-site cohort study provides information on risk factors for a severe outcome and differences in the clinical course of the respective infections.

## Conclusion

In conclusion, cancer patients tested positive for either SARS-CoV-2 or seasonal influenza had a markedly increased in-hospital mortality making them a high-risk population.

This study underlines the absolute necessity of protecting the cancer population against SARS-CoV-2 and seasonal influenza.

## Data Availability

Helios Health and Helios Hospitals have strict rules regarding data sharing because of the fact that health data are a sensible data source and have ethical restrictions imposed due to concerns regarding privacy. Access to anonymized data that support the findings of this study is available on request from the Leipzig Heart Institute (www.leipzig-heart.de).
